# Longitudinal analysis of electronic health records reveals medical conditions associated with subsequent Alzheimer’s disease development

**DOI:** 10.1186/s13195-025-01914-4

**Published:** 2025-12-29

**Authors:** Xue Zhong, Gengjie Jia, Zhijun Yin, Rui Chen, Kerou Cheng, Andrey Rzhetsky, Bingshan Li, Nancy J. Cox

**Affiliations:** 1 Division of Genetic Medicine and Clinical Pharmacology, Department of Medicine, Vanderbilt University Medical Center, Nashville, TN USA; 2 Vanderbilt Genetics Institute, Vanderbilt University Medical Center, Nashville, TN USA; 3 Department of Medicine, Institute of Genomics and Systems Biology, University of Chicago, Chicago, IL USA; 4 Department of Biomedical Informatics, Vanderbilt University Medical Center, Nashville, TN USA; 5 Department of Molecular Physiology and Biophysics, Vanderbilt University, Nashville, TN USA; 6 Department of Computer Science, Vanderbilt University, Nashville, TN USA; 7 Committee on Genomics, Genetics and Systems Biology, University of Chicago, Chicago, IL USA; 8 Department of Human Genetics, University of Chicago, Chicago, IL USA

**Keywords:** Alzheimer’s disease, Electronic health records, Longitudinal, Risk factors

## Abstract

**Background:**

Several health conditions are known to increase the risk of Alzheimer’s disease (AD). We aim to systematically identify medical conditions that are associated with subsequent development of AD by leveraging the growing resources of electronic health records (EHRs).

**Methods:**

This retrospective cohort study used de-identified EHRs from two independent databases (MarketScan and VUMC) with 153 million individuals to identify AD cases and age- and gender-matched controls. By tracking their EHRs over a 10-year window before AD diagnosis and comparing the EHRs between AD cases and controls, we identified medical conditions that occur more likely in those who later develop AD. We further assessed the genetic underpinnings of these conditions in relation to AD genetics using data from two large-scale biobanks (BioVU and UK Biobank, total *N* = 450,000).

**Results:**

We identified 43,508 AD cases and 419,455 matched controls in MarketScan, and 1,320 AD cases and 12,720 matched controls in VUMC. We detected 406 and 102 medical phenotypes that are significantly enriched among the future AD cases in MarketScan and VUMC databases, respectively. In both EHR databases, mental disorders and neurological disorders emerged as the top two most enriched clinical categories. More than 70 medical phenotypes are replicated in both EHR databases, which are dominated by mental disorders (e.g., depression), neurological disorders (e.g., sleep orders), circulatory system disorders (e.g. cerebral atherosclerosis) and endocrine/metabolic disorders (e.g., type 2 diabetes). We identified 19 phenotypes that are either associated with individual risk variants of AD or a polygenic risk score of AD.

**Conclusions:**

In this study, analysis of longitudinal EHRs from independent large-scale databases enables robust identification of health conditions associated with subsequent development of AD, highlighting potential opportunities of therapeutics and interventions to reduce AD risk.

**Supplementary Information:**

The online version contains supplementary material available at 10.1186/s13195-025-01914-4.

## Background

Alzheimer’s disease (AD) poses a huge healthcare burden in the United States and globally. AD is an age-related neurodegenerative disorder characterized by an initial decline in memory and subsequent impairment of executive and cognitive functions [1]. Until recently, there were no effective disease-modifying treatments available for AD. Since 2021, three anti-amyloid drugs have been FDA-approved that demonstrate efficacy in removing the pathological amyloid proteins and slowing cognition decline in patients with early Alzheimer’s disease (mild cognitive impairment or mild dementia due to Alzheimer’s disease) [2–4]. It is known that AD develops over decades, with the gradual accumulation of amyloid and tau proteins in the brain before the clinical manifestations and lapse in memory and cognition. Several health conditions have been linked to an increased risk of developing Alzheimer’s disease late in life. For example, midlife hypertension, hyperlipidemia, and stroke have been shown to increase the incidence of AD two decades later [5, 6]; changes in vision among adults over the age of 50 have been associated with the accumulation of two hallmark proteins of AD in the brain [7, 8]; and depression was more frequently observed in individuals who later received an AD diagnosis compared to their peers [9, 10]. While the nature of these conditions in relation to AD may vary (some are risk factors, others may serve as early manifestations), they are all associated with a higher incidence of AD. These conditions may enable earlier recognition of functional changes associated with AD as well as potential intervention long before the hallmark clinical symptoms of memory and/or cognition impairment become apparent. It is projected that delaying the onset of AD by just five years could halve the incidence rate [11].

The current inventory of medical conditions that predict the future development of AD is limited. Longitudinal electronic health records (EHRs) enable the tracking of disease progression and may reveal functional changes associated with AD before the onset of memory and/or cognition lapses. Numerous studies have established that diagnosis codes (billing codes, ICD codes) of AD within EHRs are sufficiently accurate for research purposes, with a positive predictive value of ~ 75% for AD diagnosis codes in most health systems [12–14]. We propose that longitudinal EHRs from millions of individuals can facilitate a systematic identification for medical conditions that predict the future onset of AD.

Here, we systematically mined longitudinal EHR data in MarketScan, a national-level claim-based database with over 150 million individuals, as the discovery cohort, and identified medical phenotypes that are significantly enriched in those who later receive an AD diagnosis. We analyzed the longitudinal data in VUMC’s hospital-based EHR system with about 3 million patients as an independent replication cohort to validate the findings. To further tease apart clinical phenotypes that are potential mediators of AD, we assessed the genetic basis of the robustly identified medical phenotypes through a phenome-wide association (PheWAS) [15] analysis of genetic instruments constructed using the latest genome-wide association study (GWAS) of AD and related dementia [16].

## Methods

### Data sources

The MarketScan claims database contains US national-level collection of health records for over 151 million individuals enrolled between 2003 and 2013 ^17^. This database comprises insurance claim-based records that include inpatient and outpatient medical events, procedures, medications, and healthcare expenditures. The data were sourced from numerous private insurance companies, managed care organizations, health plan providers, and state Medicaid agencies. The covered patient population therefore represent affluent, privately insured segments of US society [17, 18].

The Synthetic Derivative (SD) is the de-identified mirror of the Vanderbilt University Medical Center (VUMC) EHR database. SD contains diagnostic codes, procedure codes, hospital admissions, provider progress notes, discharge summaries, laboratory data, and medication histories. The SD data are updated regularly, and for this study, we extracted all available data till the end of January 2020 which contains EHRs from about 3 million individuals.

BioVU is the biobank of VUMC, which houses DNA samples from more than 250,000 individuals from the SD. Samples selected for genotyping are enriched for patients whose medical home is VUMC. A detailed description of program operations, ethical considerations, and continuing oversight and patient engagement has been published [19]. Genotype data from BioVU were imputed using the Haplotype Reference Consortium reference panel (version r.1.1) [20, 21] with the IMPUTE2 software [22]. For analysis, samples of European ancestry were selected based on principal component analysis (PCA) of genetic ancestry.

The included BioVU participants had a median age of 64 (IQR = [47, 75]) at their last documented ICD code, with 56% having EHR-reported sex of female. The prevalence of obesity, diabetes, hyperlipidemia and essential hypertension are 35%, 20%, 26% and 35%, respectively. In UK Biobank population [23], the average age at enrollment is 56.5 (SD = 8.0) with 54% of female. The self-reported obesity, diabetes, hyperlipidemia and essential hypertension have prevalence of 3%, 5%, 9% and 19%, respectively.

### Identify AD cases and matched controls

Previous studies have shown that the diagnosis codes of AD recorded in EHRs achieve sufficient accuracy for research purposes, with a positive predictive value of 75% for AD diagnosis codes across most health systems [12, 14]. In this study, we used the diagnosis codes of AD (ICD9: 331.0, 3310.00; ICD10: F00, F00.0, F00.1, F00.2; G30, G30.0, G30.1, G30.8, G30.9) to define AD cases. We required a minimum of two codes on separate visit dates to instantiate an AD case. We defined controls as individuals without any AD diagnosis codes in their records up to their last visit. To enhance statistical power, we matched each AD case with four to ten controls rather than using a 1:1 case/control ratio (if more than 10 controls are available to match an AD case, we randomly sampled 10 control individuals to comprise the control set for this AD case; if less than 4 controls are available to match an AD case, we do not consider that AD case nor the controls). Controls were selected per case of AD such that their age and gender are matched at the index dates. For a case of AD, the index date is the first date of AD diagnosis; for a control, the index date is the date of last recorded visit in EHRs. We required all AD cases and matched controls to maintain at least 10 years of EHRs before the index dates.

### Medical phenotype definition

We used phenotypes derived from the billing codes of electronic health records, as previously described [15], to define medical phenotypes. The resulting binary phenotypes are referred to as ‘phecodes,’ each of which may correspond to multiple diagnosis codes (ICD9 or ICD10). For example, the phecode for *dementia with cerebral degenerations* encompasses a range of ICD codes, including frontotemporal dementia (ICD9 code 331.1) and dementia with Lewy bodies (ICD9 331.82). For more details, one can download the ICD-to-phecode mapping file from https://www.vumc.org/cpm/phemap. Automatic conversion of ICD codes to pehcodes can be done using the *mapCodesToPhecodes* function from the *PheWAS* R package. Phecode has defined case, control and exclusion criteria, and we required a minimum of two codes on different visit dates to instantiate a case for each phecode.

### Statistical analysis

For each binary phenotype (‘phecode’), we compared the number of cases (unique individuals) between AD patients and matched controls over a 10-year preceding period. Fisher’s exact test (function *fisher.test* in R package “stats”) was employed to assess the relative enrichment of the associated medical event in AD patients compared to controls. We required each phecode has at least five cases in AD and non-AD groups to meet the criterion of applying the Fisher’s exact test. The analysis was performed in MarketScan and VUMC separately. At each site, we applied Bonferroni correction to determine the threshold for statistical significance. A total of 1,722 phecodes in MarketScan and 1,112 phecodes in VUMC were analyzed for enrichment. To identify disproportionately enriched clinical categories for AD-enriched phenotypes (phecodes), we compared the proportion of the enriched phecodes per clinical category to the overall proportion of enriched phecodes (i.e. overall proportion = 466/1722 = 0.27 for MarketScan and overall proportion = 102/1112 = 0.092 for VUMC) using the one-sided proportion test (function *prop.test* in R package “stats” with the parameter *alternative = “greater”*). Similarly, to identify the disproportionately depleted clinical categories for AD-depleted phenotypes, we compared the proportion of the depleted phecodes per clinical category to the overall proportion of depleted phecodes (i.e. overall_proportion = 106/1722 = 0.06 for MarketScan and overall_prop = 2/1112 = 0.0018 for VUMC).

### Sensitivity analysis

The co-occurrence of an AD diagnosis with diagnosis of other dementia types appeared common in EHRs. To assess how sensitive the enrichment estimation is affected due to such comorbidity, we constructed a sensitivity analysis in the larger database, MarketScan. Specifically, in MarketScan, we excluded AD cases who also carried diagnoses of other dementia types, including *vascular dementia*, *frontotemporal dementia*, *dementia with Lewy bodies*, *dementias*, *dementia with cerebral degeneration*, *delirium dementia and amnestic and other cognitive disorders*, and *senile dementia*. Correspondingly, we also removed the controls matched to such AD cases. This resulted in a subset with 13,391 AD cases and 133,910 matched controls involving 1644 phecodes feasible for the Fisher exact test, on which we repeated the enrichment analysis. Bonferroni correction threshold of 3.04 × 10^− 5^ (= 0.05/1644) was used to determine the significance of associations.

### Polygenic risk score

The genetic instruments we used to assess the relationship between the enriched phenotypes and AD genetics include risk variants of AD and an aggregate of these variants (as a polygenic risk score). The polygenic risk score (PRS) of AD was constructed using the APOE locus and 83 independent signals as reported from the largest GWAS study of AD and related dementia [16]. We explored the LD structure in individuals of European descent (using https://ldlink.nih.gov/?tab=ldproxy) and verified that the pairwise correlation (r^2^) between any two variants is < 0.05. These 84 risk variants will also be assessed for variant-level phenome-wide associations. The PRS is calculated as a weighted sum of the dosage of each AD-risk-increasing allele multiplied by the effect size (natural logarithm of odds ratio) of that allele. In BioVU genotyped data, there are 79 risk variants available for analysis (see Suppl. Table S1), which were used to calculate the PRS. The resulting polygenic risk score of BioVU individuals of European ancestry was used in PheWAS analysis.

### PheWAS

A systematic interrogation of phenotypic associations of genetic instrument variables of AD was performed in BioVU and UK Biobank using the PheWAS approach. In BioVU (*n* = 54000 individuals of European ancestry), PheWAS of PRS and PRS excluding the APOE locus were performed. This was achieved through logistic regression, adjusted for age, gender, three principal components of ancestry, and arrays/batches, utilizing the PheWAS package [24]. Only phecodes with at least 20 cases were included in analysis. In addition, PheWAS of the APOE locus (rs429538_C) was also performed in BioVU. The variant-level phenome-wide associations of a total of 84 AD risk variants (Suppl. Table S1) are based on the UK Biobank data and the Pheweb source (https://pheweb.sph.umich.edu) was used to extract the association results. The Pheweb displays GWAS results for ~ 1400 binary phecodes (derived from EHR ICD billing codes) across 28 million variants that were genotyped and imputed using TOPMed [25] from ~ 400,000 White British individuals of the UK Biobank. Analyses on binary outcomes were conducted using SAIGE [26] (a method of association test accounting for case-control imbalance that is typical of EHR data), adjusting for genetic relatedness, sex, birth year and the first 4 principal components.

### Time to event analysis

To further explore the longitudinal aspects of the EHR data, we conducted time to event analysis in VUMC by running Cox proportional hazards models per medical condition, with age and sex as covariates. Specifically, we set an observation window of 20 years, from 2000-01-01 (baseline) to 2020-01-01 (end of observation window), and extracted EHRs of individuals based on the following criteria. Inclusion criteria: age at baseline was 55 years old or above. Exclusion criteria: (i) the first AD diagnosis code occurred before baseline; (ii) sex is unknown. For each medical condition, we define condition = 1 if the first diagnosis code of the condition of interest occurred before the baseline, and “condition” = 0 if no such diagnosis was received or the code occurred after baseline. We set “time” as the duration from baseline to the first code of AD diagnosis or the last time of follow-up, whichever came first. We define “status” = 1 if the individual was diagnosed for AD within the observation window and “status” = 0 otherwise. We then ran cox regression for each condition with sex and baseline age as the covariates:

$$\mathrm{coxph}(\mathrm{Surv}(\mathrm{time},\;\mathrm{status})\;\sim\;\;\mathrm{condition}+\mathrm{age}+\mathrm{sex})$$

The *coxph* and *survfit* functions from R package “survival” and *ggsurvplot* function from R package “survminer” were used for analysis and visualization.

## Results

### Characteristics of study cohorts

**Fig. 1:**
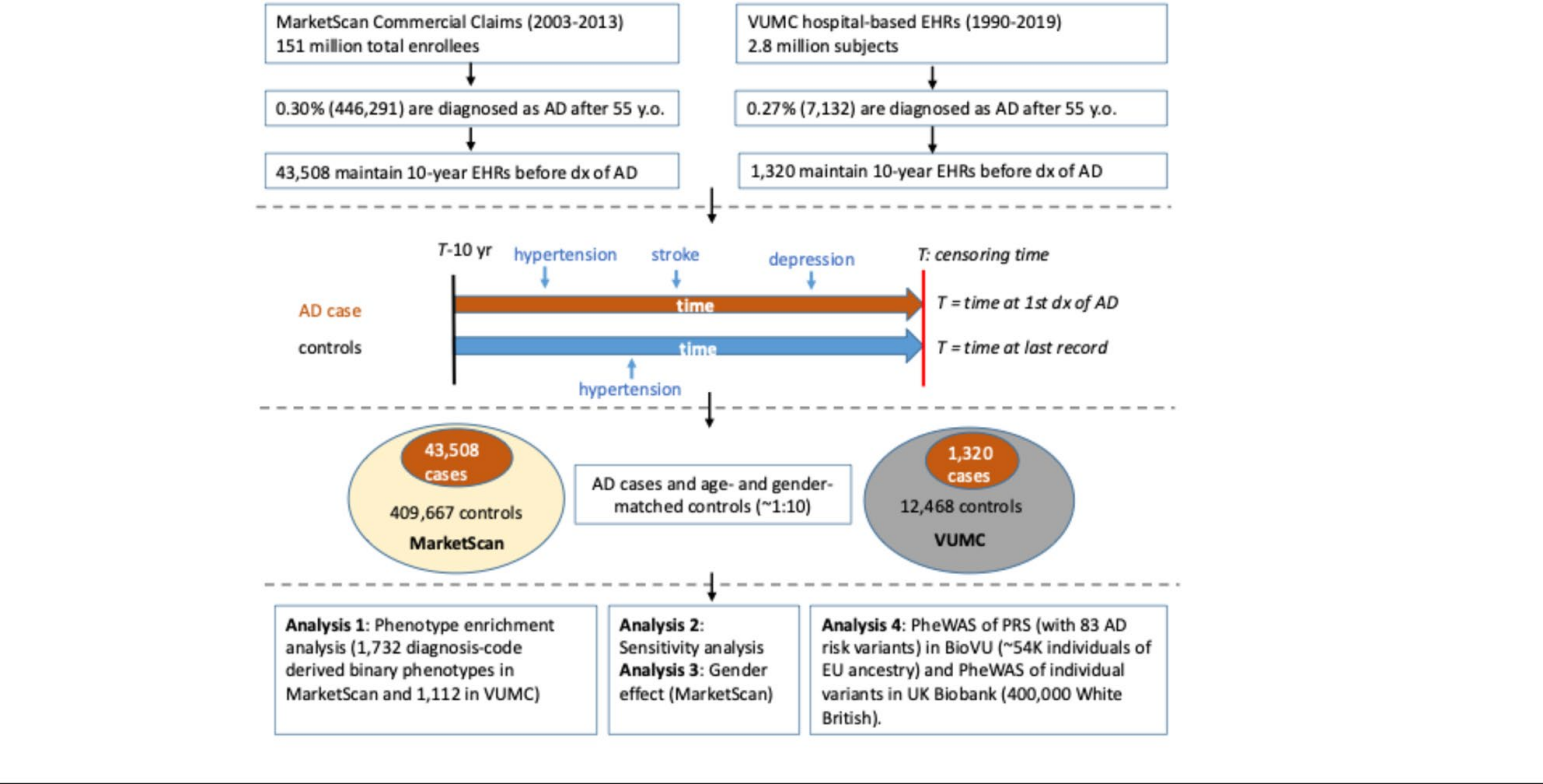
Study workflow and matching design

The workflow and study design are illustrated in Fig. 1. Utilizing the MarketScan (MS) database, a commercial, claim-based national repository of EHRs encompassing over 150 million individuals from 2003 to 2013, we identified 446,291 individuals (~ 0.3% of the population) with a diagnosis code for AD recorded after age 55. The majority (~ 90%) of these AD diagnoses, approximated by the first appearance of the AD ICD code, occurred after 65 years old. From this group, 43,508 individuals (56% female) with at least 10 years of EHRs prior to their first AD diagnosis were selected. Each AD case was then paired with 4 to 10 controls of equivalent age and gender who also had at least 10 years of EHRs prior to the index date (i.e. the date of last follow-up) and did not have an AD diagnosis code. In total, we obtained 43,508 AD cases and 419,455 matched controls from the MS database (Table 1). Using the same methodology, we identified 1,320 AD cases and 12,720 age- and gender-matched controls from the de-identified EHRs of about 3 million individuals at VUMC as of Jan 2020 (Table 1). Notably, a consistent proportion of AD cases was observed across both databases (0.30% in MS and 0.27% in VUMC), predominantly among females (56% in MS and 62% in VUMC), with the age of diagnosis predominantly above 75 years (84% in MS and 81% in VUMC) (Table 1).

**Table 1:** Characteristics of source populations and derived cohorts of AD patients and controls

Source population	MarketScan	VUMC
Description	Insurance claims-based	Hospital-based
Sample size	151 million	2.8 million
Age (yr)		
median [IQR]	35 [17, 51]	44 [23, 65]
Sex (female%)	51.3%	53.7%
Diagnosed (dx) with AD, *n*	446,291	7,132
Statify by age at diagnosis, n (%)		
< 65 y	52,579 (12%)	613 (9%)
65–74 y	86,703 (19%)	1411 (20%)
75–84 y	194,257 (44%)	3469 (49%)
>=85 y	112,752 (25%)	1639 (23%)

**Derived longitudinal cohorts of AD cases and controls**	**MarketScan**	**VUMC**
**AD dx at age 55 or older & >10y EHRs before dx**		
n (female%)	43,508 (female 56%)	1,320 (female 62%)
Age (median, IQR)	83 [78, 88]	81 [76, 85]
Stratify by age at diagnosis, *n* (%)		
55–64 y	1,859 (4%)	17 (2%)
65–74 y	5,292 (12%)	237 (18%)
75–84 y	17,431 (40%)	698 (53%)
>=85 y	18,926 (44%)	368 (28%)
**Matched Controls**		
*n* (female%)	419,455 (female 56%)	12,720 (female 62%)
Age (median, IQR)	83 [77, 88]	81 [77, 85]

*: At VUMC, any age above 90 is coded as 90 for de-identification purpose

For the identified AD cases and controls, we subsequently traced their EHRs over a 10-year period prior to an index date. For a case of AD, the index date corresponds to the date of first diagnosis of AD; for a matched control, the corresponding index date is the date of attaining the age same as that when the AD case was diagnosed. We compared the prevalence of each medical condition available in the records between the case and control groups to pinpoint conditions that occurred more often in those who later developed AD. In the two EHR databases, we employed phecodes (binary phenotypes derived from ICD billing codes) [15] to delineate the “case” and “control” status for each respective medical phenotype.

### Discovery in MarketScan

In the MarketScan database, a total of 1,722 phecodes were tested for their association with future AD cases, and 406 phecodes were significantly enriched in future AD cases compared to the matched controls after Bonferroni correction (at a significance of *P* = 0.05/1722 = 2.90 × 10^−5^). Conditions such as hypertension, hyperlipidemia, and type 2 diabetes (T2D) were more prevalent in those who later developed AD, aligning with previous observations based on prospective cohorts [6]. Memory loss (OR = 8.0, *P* < 10^− 321^) and mild cognitive impairment (OR = 6.0, *P* < 10^− 321^), two canonical clinical manifestations of AD, were among the most enriched phenotypes. This was followed by other dementia types, such as *vascular dementia* (OR = 6.4, *P* < 10^− 321^) and *dementia with cerebral degenerations* (OR = 4.8, *P* < 10^− 321^). Other highly enriched phenotypes included a range of psychological symptoms, such as *paranoid disorders* (OR = 6.3, *P* < 10^− 321^), *psychosis* (OR = 4.6, *P* < 10^− 321^), *mood disorders* (OR = 3.4, *P* < 10^− 321^), *depression* (OR = 2.4, *P* < 10^− 321^), and *transient alteration of consciousness* (OR = 2.3, *P* < 10^− 321^); neurological disorders such as *encephalopathy* (OR = 3.1, *P* < 10^− 321^), *cerebral degeneration* (OR = 2.6, *P* < 10^− 321^), *Parkinson’s disease* (OR = 2.6, *p* < 10^− 321^), *lack of coordination* (OR = 2.0, *P* < 10^− 321^), and *abnormality of gait* (OR = 1.9, *p* < 10^− 321^); as well as circulatory system issues such as *cerebral ischemia* (OR = 2.4, *P* < 10^− 321^), *cerebral artery occlusion with cerebral infarction* (OR = 1.9, *P* < 10^− 321^), and *cerebrovascular disease* (OR = 1.9, *P* = 9.0 × 10^− 172^) (see Suppl. Table S2 for the full list).

Since each phenotype can be categorized within a broader clinical domain (totaling 17 categories, see Suppl. Table S2), we then investigated whether certain clinical categories disproportionately contributed to the enrichment of phenotypes associated with AD. After assigning the phecodes to their respective clinical categories, we assessed the enrichment of each category using Bonferroni correction (at a significance of *P* = 0.05/17 = 0.0029), while considering the size of each category (for example, the *circulatory systems* category contains twice as many phenotypes as the *mental disorders* category, thereby potentially contributing to a greater number of enriched phenotypes). We found that *mental disorders*, a clinical category containing 63 phecodes, was among the most significantly enriched, with 56 of these phecodes showing enrichment (56/63 compared to the overall proportion 406/1722, proportion test *P* = 8.2 × 10^− 34^). Other top enriched clinical categories included *neurological disorders* (49/69, *P* = 3.1 × 10^− 20^), *injuries & poisonings* (60/87, *P* = 3.5 × 10^− 23^), *symptoms* (22/37, *P* = 3.7 × 10^− 7^) and *circulatory system* (53/128, *P* = 1.7 × 10^− 6^) (Suppl. Table S3). Collectively, these clinical categories, which account for 22% of the medical phenotypes, contribute to 59% (240/406) of the phenotypic enrichment observed.

Finally, we reported a list of 166 phenotypes that are depleted in the pre-AD EHRs, with a quarter of such phenotypes coming from the *neoplasms* category (Suppl. Table S4).

### Replication in VUMC

We conducted the same analysis within the VUMC database. Out of the 1,112 phecodes examined, we detected 102 significantly enriched phenotypes (at the Bonferroni correction threshold, *P* = 0.05/1112 = 4.5 × 10^− 5^) (see Suppl. Table S4). In alignment with the findings from MarketScan, *essential hypertension* (OR = 1.8, *P* = 1.2 × 10^− 23^), *hyperlipidemia* (OR = 1.6, *P* = 2.9 × 10^− 18^), and *type 2 diabetes* (OR = 1.3, *P* = 1.3 × 10^− 5^) are on the list; *memory loss* (OR = 13.3, *P* = 1.3 × 10^− 318^), *vascular dementia* (OR = 6.3, *P* = 1.4 × 10^− 44^), *depression* (OR = 3.3, *P* = 5.0 × 10^− 65^), *anxiety disorder* (OR = 2.7, *P* = 1.2 × 10^− 35^), *paranoid disorders* (OR = 8.8, *P* = 3.3 × 10^− 25^), *cerebral degeneration* (OR = 2.6, *P* = 2.8 × 10^− 13^), and *cerebral ischemia* (OR = 2.1, *P* = 1.6 × 10^− 8^) were among the most highly enriched phenotypes. The clinical categories of *mental disorders* (26/52 compared to the overall proportion 102/1112, proportion test *P* = 1.1 × 10^− 23^) and *neurological disorders* (12/50, *P* = 0.0004) also emerged as the top categories with disproportionate and significant enrichment (Suppl. Table S3). Notably, the only phenotypes that were significantly depleted were found within the *neoplasms* category (Suppl. Table S5).

### Robustly enriched phenotypes in both MarketScan and VUMC datasets

We have compiled a list of 73 phenotypes that are significantly enriched in both the MarketScan and VUMC EHR datasets after Bonferroni correction (i.e., *P* < 2.9 × 10^− 5^ in MS and *P* < 4.5 × 10^− 5^ in VUMC) (Table 2). These phenotypes, which span nine clinical categories, are predominantly represented by two: *mental disorders* (27 phecodes) and *neurological disorders* (12 phecodes). Specifically, speech disorders (*aphasia/speech disturbance*, *aphasia*, *speech and language disorder*) were more commonly seen in those who later developed AD. Severe psychological problems such as *paranoid disorders*, *psychosis*, *hallucinations* and *suicidal ideation* were also significantly enriched (all with OR > 3) over the years prior to AD diagnosis. Neurological manifestations associated with the future development of AD included *abnormal involuntary movements*,* convulsions* (involuntary muscle contractions and relaxations), *essential tremor*, and sleep disorders such as *insomnia* (inadequate sleep), *hypersomnia* (excessive sleep), and sleep-related breathing problems (*sleep apnea* and *obstructive sleep apnea*). Beyond mental and neurological disorders, the robust enrichment of phenotypes also encompasses 7 *endocrine/metabolic* phenotypes, such as *vitamin B-complex deficiencies*, *hypothyroidism* and *hypopotassemia*; 6 *circulatory system* disorders, including *essential hypertension*, *cerebral atherosclerosis*, and *cerebral ischemia*; 5 genitourinary disorders including *urinary incontinence*; 4 musculoskeletal disorders such as *osteoarthrosis* and *osteoporosis*; 3 sense organ problems including *psychophysical visual disturbances*; and 3 digestive problems such as *blood in stool* and *hemorrhage of rectum and anus*.

**Table 2:** Phenotypes significantly associated with subsequent development of AD in both EHR databases

Group	Phecode	Phecode description	Clinical category	MarketScan			VUMC		
				*P*-value	OR	%ctrl	*P*-value	OR	%ctrl
cognition	292.3	Memory loss	mental disorders	0.00*	8.0	8%	1.27E-318	13.3	6%
	292.2	Mild cognitive impairment	mental disorders	0.00	6.0	1.9%	4.55E-41	6.4	1.4%
	292.1	Aphasia/speech disturbance	mental disorders	6.7E-174	1.8	4%	9.09E-08	2.0	3%
	292.11	Aphasia	mental disorders	2.6E-99	1.9	2%	1.49E-07	2.4	2%
	315.2	Speech and language disorder	mental disorders	5.6E-19	2.3	0.2%	7.35E-06	9.0	0.1%
psychological/mood	296.2	Depression	mental disorders	0.00	2.4	14%	5.04E-65	3.3	11%
	296.22	Major depressive disorder	mental disorders	0.00	2.3	6%	2.54E-42	3.3	6%
	296.1	Bipolar	mental disorders	0.00	3.4	1.4%	1.59E-19	4.7	1.1%
	300.1	Anxiety disorder	mental disorders	2.1E-281	1.6	17%	1.10E-35	2.7	9%
	300.11	Generalized anxiety disorder	mental disorders	1.9E-100	2.7	0.6%	9.52E-09	2.6	1.7%
	300.4	Dysthymic disorder (a mild but a long-term form of depression)	mental disorders	5.8E-142	1.8	4%	1.19E-14	2.9	2.3%
	295.2	Paranoid disorders	mental disorders	0.00	6.3	0.4%	3.30E-25	8.8	0.5%
	295.3	Psychosis	mental disorders	0.00	4.6	6%	1.64E-20	3.2	2.6%
	292.4	Altered mental status	mental disorders	0.00	3.6	14%	1.12E-51	3.1	10%
	292.6	Hallucinations	mental disorders	3.7E-101	3.5	1.0%	7.87E-08	3.4	0.7%
	297.1	Suicidal ideation	mental disorders	1.5E-93	4.3	0.2%	2.84E-08	5.6	0.3%
	295.1	Schizophrenia	mental disorders	0.00	4.8	0.3%	2.67E-05	3.5	0.4%
dementia	290.1	Dementias	mental disorders	0.00	13.4	8%	0.00	22.2	7%
	290.13	Senile dementia	mental disorders	0.00	6.4	4%	5.13E-73	6.8	2%
	290.16	Vascular dementia	mental disorders	0.00	6.0	1.4%	1.43E-44	6.3	2%
	290.12	Dementia with cerebral degenerations	mental disorders	2.1E-106	6.6	1.0%	6.33E-15	5.2	0.6%
	290.2	Delirium due to conditions classified elsewhere	mental disorders	0.00	3.4	4%	3.87E-20	2.7	4%
	290.3	Other persistent mental disorders due to conditions classified elsewhere	mental disorders	0.00	7.5	6%	1.11E-82	5.9	4%
other mental disorders	291.8	Alteration of consciousness	mental disorders	0.00	2.4	9%	8.11E-17	2.2	6%
	291.4	Specific nonpsychotic mental disorders due to brain damage	mental disorders	2.4E-20	3.2	0.6%	4.26E-10	4.7	0.5%
	292.5	Transient alteration of awareness	mental disorders	0.00	2.3	4.9%	2.13E-08	3.2	0.9%
	317.1	Alcoholism	mental disorders	1.8E-86	2.1	1.2%	2.60E-08	3.2	0.9%
brain	348.8	Encephalopathy, not elsewhere classified	neurological	0.00	3.1	6%	6.18E-22	3.1	3%
	348.9	Other conditions of brain, NOS	neurological	0.00	2.2	4%	7.28E-09	1.9	5%
	331.9	Cerebral degeneration, unspecified	neurological	0.00	2.6	9%	2.84E-13	2.3	4%
	331.1	Hydrocephalus	neurological	1.7E-153	3.0	0.8%	2.91E-07	3.0	0.9%
sleep	327.4	Insomnia	neurological	2.4E-58	1.3	10%	1.62E-14	2.0	7%
	327.32	Obstructive sleep apnea	neurological	5.2E-11	1.1	10%	1.21E-11	1.9	6%
	327.3	Sleep apnea	neurological	9.6E-11	1.2	8%	1.20E-07	1.9	4%
	327.1	Hypersomnia	neurological	1.8E-17	1.4	1.4%	1.01E-05	2.7	0.8%
movement	345.3	Convulsions	neurological	0.00	2.5	4%	7.00E-14	2.4	4%
	333.1	Essential tremor	neurological	1.3E-53	1.5	3%	6.40E-10	2.3	3%
	350.1	Abnormal involuntary movements	neurological	3.8E-96	1.5	5%	2.09E-08	2.0	4%
	350.3	Lack of coordination	neurological	0.00	2.0	8%	2.80E-06	2.0	2%
endocrine/metabolic	261.2	Vitamin B-complex deficiencies	endocrine/metabolic	2.1E-169	1.6	9%	9.15E-23	2.8	4%
	244.4	Hypothyroidism NOS	endocrine/metabolic	1.1E-06	1.1	31%	1.11E-15	1.7	16%
	276.5	Hypovolemia	endocrine/metabolic	0.00	1.7	17%	2.29E-12	1.7	13%
	276.14	Hypopotassemia	endocrine/metabolic	1.9E-117	1.4	13%	1.22E-08	1.6	12%
	250.24	Type 2 diabetes with neurological manifestations	endocrine/metabolic	9.1E-48	1.3	9%	6.03E-07	1.7	6%
	251.1	Hypoglycemia	endocrine/metabolic	7.8E-58	1.5	4%	5.20E-06	2.3	1.4%
	250.2	Type 2 diabetes	endocrine/metabolic	6.8E-35	1.1	36%	1.26E-05	1.3	23%
circulatory system	401.1	Essential hypertension	circulatory system	4.8E-71	1.3	87%	1.24E-23	1.8	60%
	433.12	Cerebral atherosclerosis	circulatory system	4.3E-98	1.7	3%	8.46E-14	2.6	3%
	433.8	Late effects of cerebrovascular disease	circulatory system	0.00	1.9	8%	5.66E-09	1.9	5%
	433.3	Cerebral ischemia	circulatory system	0.00	2.4	8%	1.63E-08	2.1	3%
	401.21	Hypertensive heart disease	circulatory system	9.1E-25	1.1	16%	5.82E-07	1.4	17%
	433.31	Transient cerebral ischemia	circulatory system	0.00	1.7	17%	3.50E-06	1.6	6%
genitourinary	599.4	Urinary incontinence	genitourinary	2.6E-206	1.5	14%	8.63E-19	2.1	9%
	599.5	Frequency of urination and polyuria	genitourinary	3.6E-85	1.3	19%	3.16E-18	2.0	11%
	599.9	Other abnormality of urination	genitourinary	8.9E-26	1.2	7%	3.63E-08	1.8	5%
	585.1	Acute renal failure	genitourinary	7.9E-64	1.7	2%	6.46E-06	1.4	13%
	624.9	stress incontinence, female	genitourinary	7.2E-08	1.1	4%	2.48E-05	1.9	2%
musculoskeletal	740.9	Osteoarthrosis NOS	musculoskeletal	1.6E-50	1.2	49%	4.80E-29	2.0	19%
	741.3	Difficulty in walking	musculoskeletal	3.1E-254	1.5	17%	8.01E-20	2.5	5%
	743.11	Osteoporosis NOS	musculoskeletal	6.9E-34	1.2	24%	1.83E-15	1.8	12%
	721.1	Spondylosis without myelopathy	musculoskeletal	4.7E-10	1.2	2%	3.17E-11	1.6	14%
symptoms	772.3	Muscle weakness	symptoms	0.00	1.8	16%	1.53E-17	2.0	9%
	782.3	Edema	symptoms	2.3E-13	1.1	33%	1.17E-12	1.6	18%
	771.1	Swelling of limb	symptoms	2.9E-71	1.2	22%	2.11E-07	1.7	7%
sense organ	380.4	Impacted cerumen	sense organs	1.0E-06	1.1	24%	5.97E-11	1.8	8%
	379.9	Pain, swelling or discharge of eye	sense organs	6.5E-06	1.1	4%	1.18E-07	2.7	1%
	368.91	Psychophysical visual disturbances	sense organs	1.5E-76	3.2	0.3%	1.54E-07	4.3	0.4%
	561.1	Diarrhea	digestive	1.0E-76	1.3	19%	4.57E-17	1.9	11%
digestive	578.8	Hemorrhage of rectum and anus	digestive	3.2E-09	1.1	12%	6.62E-08	2.1	3%
	578.2	Blood in stool	digestive	1.5E-11	1.1	11%	2.74E-06	1.7	5%
other	512.8	Cough	respiratory	6.8E-19	1.1	48%	1.75E-24	1.9	22%
	110.11	Dermatophytosis of nail	infectious diseases	1.2E-321	1.5	29%	5.56E-15	2.2	6%
	803.2	Fracture of radius and ulna	injuries & poisonings	8.0E-40	1.3	5%	1.20E-06	2.2	2%

*p-value less than 2.2E-308 is reported as 0

%ctrl: the prevalence of a phenotype in non-AD individuals

### A portion of the enriched phenotypes are genetically linked to AD

Having identified the phenotypes that are reliably detected in two independent large-scale EHR databases and are enriched in individuals at risk for future AD, we are now investigating the genetic underpinnings of these phenotypes. Phenotypes that share genetic susceptibility with AD and precede the clinical onset of AD may serve as early biomarkers before the irreversible cognition lapses. We compiled AD risk loci from the latest GWAS of AD and related dementia (ADRD) [16] and constructed an instrumental variable by aggregating the independent ADRD risk alleles weighted by their effect size. The resulting polygenic risk score, PRS_AD_, consists of 78 independent signals including the APOE locus (Suppl. Table S1). Using the genotype data of European ancestry from BioVU (*n* = 54,594), we calculated the PRS_AD_ for each person and performed PheWAS analysis across 1,493 phecodes. This analysis revealed 17 significant associations (at Bonferroni correction *P* < 0.05/1493 = 3.3$\:\times\:$10^−5^), including 5 phenotypes inversely associated with PRS_AD_. *Alzheimer’s disease* (*P* = 7.3 × 10^− 42^), *memory loss* (*P* = 2.5 × 10^− 20^), and *mild cognitive impairment* (*P* = 5.2 × 10^− 15^) are positively associated with PRS_AD_. *Dementias* (*P* = 6.5 × 10^− 50^) and subtypes such as *delirium dementia and amnestic and other cognitive disorder* (*P* = 3.1 × 10^− 32^), *senile dementia* (*P* = 1.6 × 10^− 7^), and *vascular dementia* (*P* = 2.0 × 10^− 7^) are also among the significant and positive associations. Other positive associations include *disorders of lipoid metabolism* (*P* = 5.6 × 10^− 8^), *hyperlipidemia* (*P* = 7.4 × 10^− 8^), *hypercholesterolemia* (*P* = 1.1 × 10^− 7^), and *neurological disorders* (*P* = 1.5 × 10^− 6^). Focusing on the phenotypes positively associated with PRS_AD_, we identified 9 such phenotypes that are not only showed enrichment prior to AD diagnosis but also are genetically linked to AD (Table 3). Upon excluding the APOE e4 allele (i.e. rs429358_C) from the PRS_AD_ and repeating the association analysis, none of the previously significant associations retained their significance (although nominal significance was observed for several phenotypes, such as *Alzheimer’s disease* (*P* = 0.0009), *dementias* (*P* = 0.0002), and *senile dementia* (*P* = 0.005)) (Suppl. Table S6). This finding suggests that the observed phenotypic associations with PRS_AD_ are largely attributable to the APOE locus.

**Table 3:** Phenotypes supported by AD genetics

Category	Phenotype	Genetic instrument*	*P*-value	Biobank
mental disorders	Dementias	APOE_rs429358_C	1.60E-91	UKBB
		PRS	6.50E-50	BioVU
	Delirium dementia and amnestic and other cognitive disorders	APOE_rs429358_C	6.40E-67	UKBB
		PRS	3.09E-32	BioVU
	Vascular dementia	APOE_rs429358_C	1.00E-17	UKBB
		PRS	1.98E-07	BioVU
	Altered mental status	APOE_rs429358_C	7.50E-12	UKBB
	Neurological disorders	APOE_rs429358_C	8.10E-12	UKBB
		PRS	1.45E-06	BioVU
	Memory loss	PRS	2.5E-20	BioVU
	Mild cognitive impairment	PRS	5.2E-15	BioVU
	Other persistent mental disorders due to conditions classified elsewhere	PRS	8.8E-10	BioVU
	Delirium due to conditions classified elsewhere	APOE_rs429358_C	2.10E-08	UKBB
	Senile dementia	PRS	1.59E-07	BioVU
endocrine/metabolic	Hyperlipidemia	PRS	7.40E-08	BioVU
		APOE_rs429358_C	1.4E-68	UKBB
	Disorders of lipoid metabolism	PRS	5.60E-08	BioVU
		APOE_rs429358_C	2.7E-68	UKBB
	Hypercholesterolemia	PRS	1.10E-07	BioVU
		APOE_rs429358_C	3.2E-66	UKBB
circulatory system	Coronary atherosclerosis	APOE_rs429358_C	2.00E-22	UKBB
	Ischemic Heart Disease	APOE_rs429358_C	3.60E-17	UKBB
	Hypertension	SPI1_rs10437655_A	2.40E-12	UKBB
		MME_rs61762319_A	1.20E-06	UKBB
	Essential hypertension	SPI1_rs10437655_A	4.10E-12	UKBB
		MME_rs61762319_A	1.10E-06	UKBB
	Other chronic ischemic heart disease, unspecified	APOE_rs429358_C	5.80E-10	UKBB
	Cerebral ischemia	APOE_rs429358_C	1.20E-05	UKBB
	Cerebrovascular disease	APOE_rs429358_C	2.90E-05	UKBB
neurological	Other cerebral degenerations	APOE_rs429358_C	2.90E-05	UKBB

*In each genetic instrument, the individual AD risk allele or the count of risk alleles (PRS) are presented

In addition to PRS_AD_, we also assessed the variant-level phenotypic associations using a larger biobank (UK Biobank) with 400,000 genotyped white British samples. We interrogated the phenome-wide associations and yielded 82 significant associations (*P* < 0.05/1400 = 3.6 × 10^− 5^) involving 17 AD risk loci (Suppl. Table S7). The AD risk allele at the APOE locus (rs429358_C) is positively associated with 17 phenotypes, recapitulating the phenotypes seen for the associations with PRS_AD_. These include *Alzheimer’s disease* (*P* = 4.3 × 10^− 66^), *dementias* (*P* = 1.6 × 10^− 91^), *vascular dementia* (*P* = 1.0 × 10^− 17^), *hyperlipidemia* (*P* = 1.4 × 10^− 68^), *disorders of lipoid metabolism* (*P* = 2.7 × 10^− 68^), and *hypercholesterolemia* (*P* = 3.2 × 10^− 66^). In addition to mental and endocrine/metabolic disorders, the APOE e4 risk allele was also associated with an increased risk of circulatory system disorders, such as *coronary atherosclerosis* (*P* = 2.0 × 10^− 22^), *ischemic heart disease* (*P* = 3.6 × 10^− 17^), *cerebral ischemia* (*P* = 3.2 × 10^− 5^), and *cerebrovascular disease* (*P* = 2.9 × 10^− 5^). *Hypertension* was associated with two other AD risk alleles, located at the SPI1 locus (rs10437655_A) and MME locus (rs61762319_A), respectively. In total, there are 22 unique phenotypes that are robustly associated with a higher risk of AD are supported by AD genetics, through either PRS or individual risk alleles, or both (Table 3).

### Gender effect

Given that women are at a higher risk of developing AD than men, we were curious to determine whether any of the phenotypic enrichment observed was more pronounced in one gender over the other. For this purpose, we stratified the MS samples by gender and re-estimated the enrichment for each gender. To ensure a fair comparison, we focused on general phenotypes by excluding male-only and female-only phenotypes (medical conditions involving sexual organs). Overall, of the phenotypes that reached nominal significance in both genders (*n* = 286), no pronounced gender effect was detected: the resulting enrichment estimates were consistent between the genders (regression line: male = 1.0334×female + 0.0243 for the natural logarithm of the ORs, correlation = 0.893) (Suppl. Table S8, Suppl. Fig S2). This indicates that our matching approach has effectively mitigated potential gender bias, and the resulting odds ratio estimates reflect a gender-independent enrichment.

### Sensitivity analysis

#### Co-occurrence of AD with other dementias and its impact on enrichment estimation

It is well documented that AD can present along with other dementia types [27–30]. We confirmed this phenomenon of co-occurrence of AD and other dementias in EHRs. For example, in MarketScan, approximately 60% of individuals diagnosed with AD had previously been diagnosed with another type of dementia. To investigate whether such co-occurrence may have influenced the enrichment estimation, we conducted an analysis within the larger MS database. Specifically, we excluded AD cases that had any prior diagnosis of other dementia (*dementias*, *senile dementia*, *vascular dementia*, *dementia with cerebral degeneration*, *delirium dementia and amnestic*,* and other cognitive disorders*) (*n* = 20,153) (Suppl. Table S9). With exclusion of these AD cases, their matched controls were also excluded from analysis (*n* = 251,030) (Suppl. Table S9). The re-estimated odds ratios from this subset of data revealed a reduction in associated phenotypes (after Bonferroni correction, *P* < 2.9 × 10^− 5^) (Suppl. Fig. S2A-B). Mental disorders are markedly reduced to half or less of their original values, for example, *delirium due to conditions classified elsewhere* (new OR = 2.0 vs. original 4.0), *developmental delays and disorders* (OR = 2.1 vs. original 5.4), *paranoid disorders* (OR = 3.5 vs. original 7.4), *psychosis* (OR = 2.6 vs. original 5.1), *conduct disorders* (OR = 3.1 vs. original 7.4), and *suicidal ideation* (OR = 2.3 vs. original 4.6). In contrast, the hallmark manifestations of AD had little changes in the resulting estimates: *memory loss* (OR = 9.1 vs. original 9.5) and *mild cognition impairment* (OR = 6.3 vs. original 7.3). This suggests that the inclusion or exclusion of AD cases with comorbidity of other dementias did not significantly affect the estimates of AD-specific manifestations, while the psychological symptoms that were heavily affected are more generic to all dementia types. Finally, we note that there was no difference in the prevalence of medical conditions observed in the control samples between the original and subset datasets (Suppl. Fig. S2C), which reassures us that the observed attenuation in estimates is not due to the comparison of the full versus subset control samples.

### Time to event analysis

Our enrichment analysis uncovered medical conditions potentially associated with AD development but lacked the resolution on temporal aspects of these conditions to AD onset. We obtained a cohort of 340,049 individuals (female 53.4%) from VUMC using inclusion/exclusion criteria and a 20-year observation window (Methods). We conducted time to event analysis (Methods), focusing on phenotypes that show robust enrichment over the years before AD onset. Of the 73 conditions being tested, we identified 20 conditions nominally associated (i.e. *P* < 0.05) with AD risk, all with hazard ratios > 1 (Suppl. Table S11), including anxiety, type 2 diabetes and sleep apnea. Seven phenotypes survived stringent Bonferroni correction (Suppl. Table S11), including hypertension (*P* = 5.7 × 10^− 25^) and hypertensive heart disease (*P* = 2.9 × 10^− 8^), which confirmed vascular contributions to AD risk. Survival plots revealed some baseline conditions associated with faster progression to AD than others. For example, the hazard ratio of *hypertensive heart disease* (HR = 5.1) was twice of that of *hypertension* (HR = 2.5) (Suppl. Fig. S3). These observations provide additional information on the temporal aspects of the enriched medical conditions in relation to AD development.

## Discussion

EHR data, a rapidly expanding yet underutilized resources, holds significant potential for AD research. With access to longitudinal EHRs from over 150 million individuals, we possess the statistical power for a systematic interrogation of medical conditions that are more likely to occur in individuals who later develop AD. By comparing the documented prevalence of medical conditions between AD patients in later life and age- and gender-matched controls over an equivalent observation period, we identified and validated 73 medical phenotypes that showed enrichment prior to AD diagnosis in both the MarketScan and VUMC databases. Among these phenotypes, mental disorders comprise one-third of the total, neurological disorders alone comprise one-sixth, endocrine/metabolic and circulatory disorders together make up another one-sixth, and the remaining third is represented by nine distinct clinical categories. Our genetic analysis further revealed connections between a small portion of these phenotypes and AD genetics, showing associations with specific AD risk variants or PRS.

Our study confirms hypertension and hypercholesterolemia as risk factors for the development of late-life AD. Without cure for dementia (the current FDA-approved amyloid-based treatment for AD is intended for patients with early/mild Alzheimer’s disease), risk reduction and prevention are of paramount importance. Modifying these risk factors through the adoption of healthier lifestyles or the use of anti-hypertensive or lipid-lowering medications that are more brain-healthy can be effective measures for risk reduction. Ongoing clinical trials for AD and related dementia are investigating several anti-hypertensives (telmisartan, losartan, amlodipine and perindopril) and lipid-lowering medication (atorvastatin) [31]. Findings from these clinical trials will provide evidence on the protective effects of these medications against dementia and will guide the selection of the most appropriate interventions.

Previous studies have noted the absence of a significant association between overall AD genetics and neuropsychiatric diseases. A study of genetic sharing among 25 brain disorders revealed extremely limited extent of genetic correlation between AD and psychiatric disorders (e.g., anxiety and major depressive disorder) as well as neurodegenerative disease (e.g., Parkinson’s disease) [32]. In a GWAS of AD involving 71,880 clinically diagnosed AD cases and proxies, along with 383,378 controls, the genetic correlation between AD and depression barely reached significance [33]. Despite the limited genetic sharing at the global scale between AD and psychiatric diseases, at individual genetic loci, some are indeed found to be shared between AD and psychiatric disorders [34, 35]. Local genetic correlation analysis from a recent study [36] further revealed four GWAS loci (e.g. the TMEM106B locus at chr7 or rs13237518) that are shared between depression and AD, five loci shared between schizophrenia and AD, and two loci shared between bipolar and AD. Therefore, among individuals with depression, bipolar, or schizophrenia, those who carry AD-causing variants may be more susceptible to developing AD than others.

An intriguing observation is that neoplasms consistently emerge as depleted phenotypes in both EHR datasets. This observation is consistent with the inverse relationship between cancer diagnosis and Alzheimer’s disease reported recently, a trend supported by accumulating evidence [37–41]. It has been hypothesized that age-related alterations in metabolism and energy balance may concurrently increase the risk of AD while lowering the risk of cancer, or vice versa [38, 42, 43]; however, the exact underlying mechanisms remain elusive. The situation is further complicated by the fact that cognitive impairment, often referred to as “brain fog,” has been reported as an adverse effect associated with cancer treatment [44].

The study has several limitations. The use of the first recorded diagnosis code of AD in the EHRs may not faithfully capture the actual date of AD onset, as the actual emergence of AD could have been earlier. Nonetheless, we observed in both EHR datasets that 80% of the AD patients received their first diagnosis code of AD at or above the age of 75, which aligns with the typical age distribution for late-onset AD. Second, we only used diagnosis codes (ICD) to identify case/control status of AD, which may result in under-detection of AD cases. The integration of medication and clinical notes may enhance the detection of AD cases [45]. In both EHRs datasets, the identified AD patients comprise approximately 0.30% of the total population, a figure lower than the national prevalence of AD cases, which is about 2% when considering the estimated 6.7 million Americans living with AD out of a total US population of 335 million as of 2023. The under-detection of AD cases may result in the inclusion of “unrecognized AD cases” within the control group, leading to a conservative attenuation in the enrichment estimation between AD and controls. Consequently, the current list of enriched phenotypes is likely to remain robust, if not more pronounced.

## Conclusion

In conclusion, our systematic interrogation of two independent large-scale EHR databases with 153 million individuals demonstrates the feasibility of utilizing the growing resources of longitudinal EHRs to substantially expand the list of health conditions for association with subsequent development of AD. By tracking and comparing the documented medical conditions over an equivalent observation period prior to AD diagnosis, we discovered and replicated more than 70 medical phenotypes that occurred more likely in those who later received AD diagnosis compared to their age- and gender-matched counterparts. Our findings reveal that the associated phenotypes are dominated by neuropsychological, circulatory and endocrine/metabolic disorders. Furthermore, about a quarter of the phenotypes demonstrate sharing with AD genetics. Together, our findings highlight potential opportunities of therapeutics and interventions to reduce AD risk.

## Supplementary Information

Supplementary Material 1

Supplementary Material 2

## Data Availability

All data generated or analyzed during this study are incorporated in this published article and its Supplementary Information. Access to the genotype and phenotype data from the UK Biobank (https://www.ukbiobank.ac.uk) and BioVU (https://victr.vumc.org/how-to-use-biovu/) requires institutional approval and compliance with a data use agreement. The variant-level phenome results based on the UK Biobank data are available at https://pheweb.sph.umich.edu. PheWAS mapping can be find at PheWAS sources (https://phewascatalog.org/phewas/).

## Abbreviations

AD: Alzheimer’s disease
ADRD: Alzheimer’s disease and related dementia
BioVU: Biobank of Vanderbilt University
EHR: Electronic health records
GWAS: Genome-wide association study
ICD9: International classification of disease version 9
ICD10: International classification of disease version 10
OR: Odds ratio
PCA: Principal component analysis
PheWAS: Phenome-wide association study
PRS: Polygenic risk score
SD: Synthetic Derivative
VUMC: Vanderbilt University Medical Center
