# Supplementary figures and images for: Longitudinal analysis of electronic health records reveals medical conditions associated with subsequent Alzheimer’s disease development
Source: Alzheimers Res Ther. 2025 Dec 29;17:263. doi: 10.1186/s13195-025-01914-4 (PMC12751976; doi:10.1186/s13195-025-01914-4)

Figure S1.

A

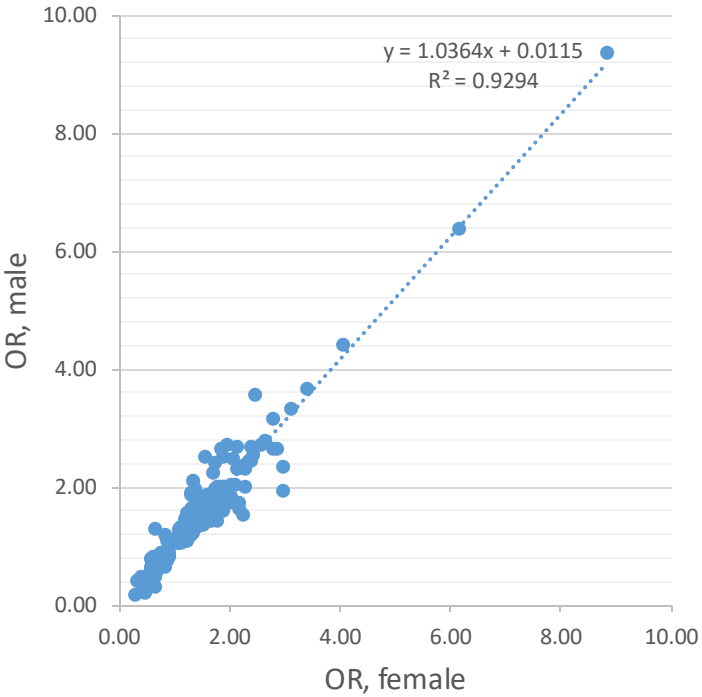

B

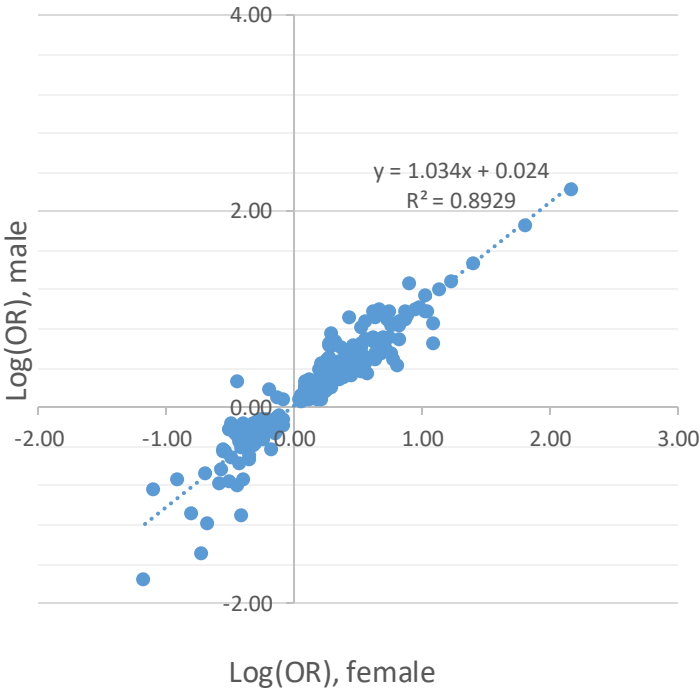

Figure S2.

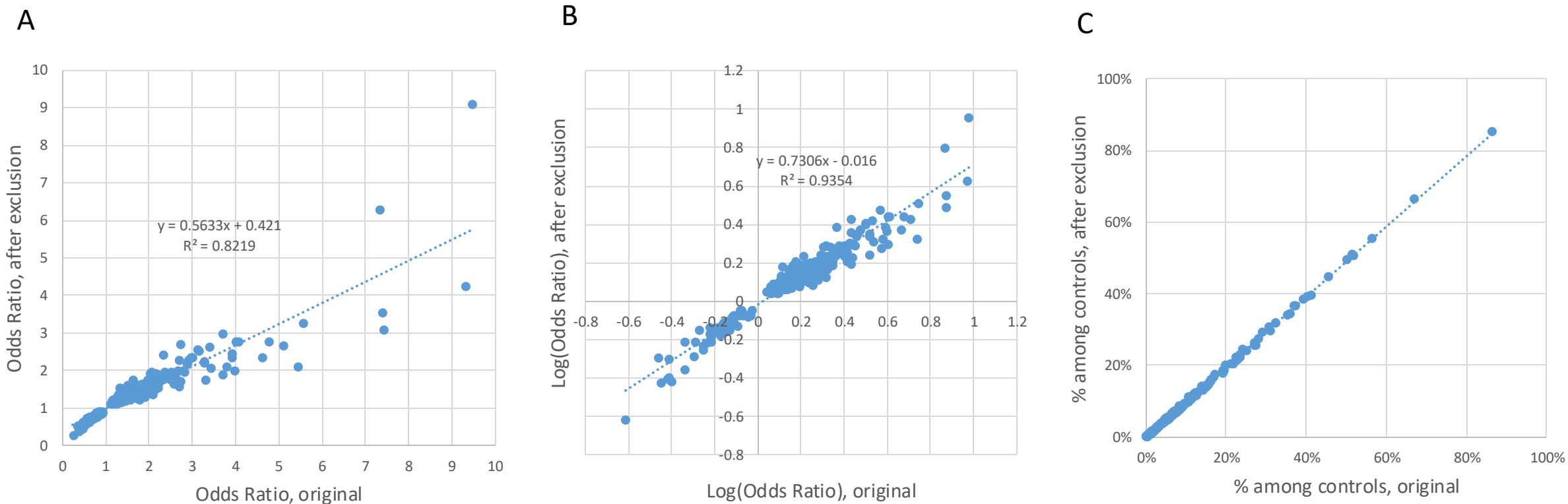

Figure S3

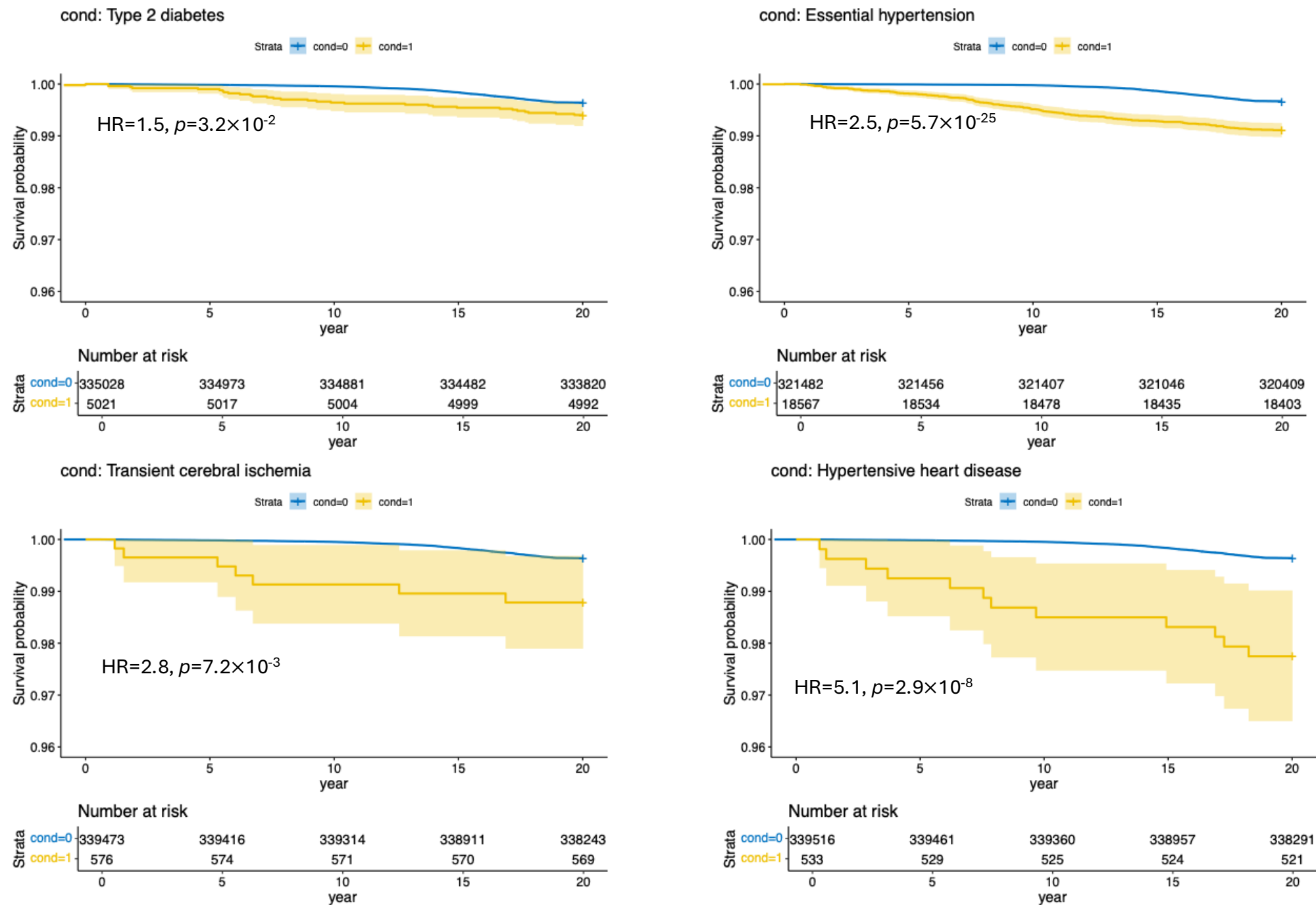

Supplement: Supplementary file 1 — Supplementary Material 1 [file 13195_2025_1914_MOESM1_ESM.pdf]
